# Non-linear effects on the population performance of Bighead Carp under different maturation schedules

**DOI:** 10.1007/s10530-023-03126-z

**Published:** 2023-08-12

**Authors:** Erik K. Dean, D. Andrew R. Drake, Nicholas E. Mandrak

**Affiliations:** 1 Department of Physical and Environmental Sciences, University of Toronto Scarborough, 1265 Military Trail, Toronto, ON M1C 1A4 Canada; 2 Great Lakes Laboratory for Fisheries and Aquatic Sciences, Fisheries and Oceans Canada, 867 Lakeshore Rd., Burlington, ON L7S 1A1 Canada; 3 Department of Biological Sciences, University of Toronto Scarborough, 1265 Military Trail, Toronto, ON M1C 1A4 Canada

**Keywords:** Population growth, Life-history strategies, Xenocyprididae, Asian carps, Invasive carps, Invasion, Laurentian Great Lakes

## Abstract

Bighead Carp currently threatens to invade the Laurentian Great Lakes from the Mississippi River, but the novel climatic conditions it will encounter by expanding northwards could affect its population performance. Bighead Carp in colder climates exhibits slower growth and matures later, with later maturation typically leading to larger adult size and increased fecundity and survival. Accordingly, the life-history strategies of Bighead Carp at its northern range limits could differ from those observed in its current invaded range. To explore how population performance could differ across changing environmental conditions, we used a stage- and age-based matrix population model parameterized with values reported for Bighead Carp populations around the world. The model was used to evaluate how different ages of maturity and their resulting impacts to body size, survival, and fecundity could impact rates of population growth and establishment. Age of maturity had a non-linear effect on population growth, with maturation at intermediate ages (4–6 years) resulting in better performance. However, performance differed less between maturation ages when fecundity was allowed to increase disproportionately with body size. Greater population growth at younger ages of maturity suggest that invasion at lower latitudes could enable establishment in fewer years due to faster rates of development in warmer temperatures. Across all maturation schedules, population growth was most sensitive to the recruitment of age-1 individuals and least sensitive to adult survival, and vital rates overall varied more in their contribution to population growth at younger ages of maturity. Thus, understanding the factors that control age-1 recruitment would inform projections of population performance for Bighead Carp in the Laurentian Great Lakes.

**Supplementary Information:**

The online version contains supplementary material available at 10.1007/s10530-023-03126-z.

## Introduction

Bighead Carp (*Hypophthalmichthys nobilis*) is a large, planktivorous fish used globally for aquaculture and biocontrol due to its ability to efficiently consume plankton and convert it into fish biomass (Jennings 1988). Like other invasive carps in the family Xenocyprididae, which includes Grass Carp (*Ctenopharyngodon idella*), Silver Carp (*Hypophthalmichthys molitrix*), and Black Carp (*Mylopharyngodon piceus*), Bighead Carp is native to eastern Asia and was first introduced to the United States in the 1970s for the purposes of biocontrol (Kolar et al. 2007; Mandrak and Cudmore 2004), but escaped captivity shortly thereafter. It has since widely dispersed throughout the Mississippi River and continues to expand its range (Currie et al. 2011; USGS NAS 2021). Having dispersed northward through the United States, Bighead Carp is now likely to enter the Laurentian Great Lakes through physical connections or human-mediated introductions (Cudmore et al. 2012). Establishment of Bighead Carp in the Great Lakes could alter food webs (Cudmore et al. 2012; Alsip et al. 2019, 2020) and threaten economically important commercial and recreational fisheries (Hayder 2014; Lauber et al. 2016). As populations of Bighead Carp expand northward, they will experience colder conditions that may affect their growth and maturation. In colder climates, Bighead Carp individuals grow at slower rates and mature later in life (Cudmore et al. 2012) compared to populations in warmer conditions (Jennings 1988). However, when individuals mature at an earlier age in warmer temperatures they do so at a smaller average size (Kolar et al. 2007). Bighead Carp reaches maturity in 3–4 years at an average weight of 3–7 kg in tropical and subtropical climates, compared to 6–8 years and 5–10 kg in temperate climates (Woynarovich and Horváth 1980). This difference is the result of how organisms allocate energy between growth and reproduction following maturation, as gonadal maintenance and gamete production costs can leave a reduced proportion of energy available for growth (Nisbet et al. 2000). The highest growth rates should occur prior to maturity, as seen in the lower Missouri river, where Bighead Carp matures as early as age 3 and growth increments peaked between the ages of 2 and 3 (Schrank and Guy 2002). Similar reductions in growth rate after maturation have been reported in Silver Carp (Williamson and Garvey 2005).

While higher temperatures may increase growth, the early onset of maturation shortens the window for elevated rates of pre-maturity growth, resulting in smaller adult size-at-age compared to those achieved under slower rates of growth maintained over a larger number of years. Smaller adult sizes are associated with a non-linear reduction in fecundity for fishes (Barneche et al. 2018) and reduced rates of survival (Lorenzen 1996). Conversely, prolonging maturation increases the length of time spent risking mortality before first reproduction, and faster growth has a greater impact on Bighead Carp survival earlier in life than later (Cuddington et al. 2014). Early maturity presents a trade-off between reproductive opportunity and lifetime reproductive output. Individuals that mature earlier in life tend to have shorter adult lifespans (Charnov 1993). Increased fecundity at younger ages is also associated with reduced lifetime fecundity (Roff 1993). For Bighead Carp, earlier maturation results in greater subadult survival, but lower adult survival and fecundity as well as a shorter lifespan. Together, these characteristics reduce the total number of reproductive years. Matrix modelling has been used to examine how Bighead Carp populations may grow under varying ages of maturity (Cuddington et al. 2014), but a constant adult size across maturation schedules was assumed. Without considering the trade-offs expected for size, survival, and fecundity, early maturation would otherwise enable rapid growth without compromise. Other studies have explored the potential effects of resource availability on growth (Cooke and Hill 2010) and temperature conditions on macrophyte consumption (van der Lee et al. 2017) for invasive xenocypridid carp populations in the Laurentian Great Lakes using the Wisconsin bioenergetics model (Hanson et al. 1997). This model also assumes a single adult body size and does not consider the variability in growth and maturation that can occur under different environmental conditions. We consider the counterbalance between vital rates and life stages that arise from maturation at different ages and explore how the opportunity cost of delayed reproduction trades off with increased adult body size, survival, and fecundity.

Using a flexible age and stage-based matrix model, we compare the performance (population growth, time until establishment) of simulated Bighead Carp populations maturing at ages ranging from 3 to 8 years. We also explore how reductions in fecundity, resulting from maturation at smaller sizes as suggested by Barneche et al. (2018), could affect populations by comparing linear and non-linear trends in fecundity across maturation ages. By parameterizing this model with life-history data reported for populations globally, we review performance across a range of conditions associated with maturation age and, thus, explore how variation in life history may affect the performance of introduced populations. Ultimately, these objectives are critical to understand how Bighead Carp could perform in new environments, including under climate change where warmer thermal conditions could hasten maturation rates, and provide insight into how the control of Bighead Carp may differ based on life history.

## Methods

### Population model

Population growth of Bighead Carp was modelled using an age- and stage-structured matrix model (Eq. 1), with a pre-breeding census, and an annual timestep. The model can accommodate a range of maturation schedules with age at first maturity between ages 3 and 8, which was selected based on the availability of published growth data. The model used recruitment, adult mortality, and fecundity parameters that varied depending on the maturation schedule considered (Supplementary Table 6). Three stages corresponding to the life history of Bighead Carp were used (Supplementary Table 7), young-of-year ($$Y$$), subadult ($$S{A}_{n}$$), and adult ($${A}_{n}$$). The young-of-year stage included post-larval age-0 fish—eggs and larvae did not occupy a distinct stage as they were incorporated into young-of-year recruitment ($${r}_{Y})$$. The subadult stage included non-reproductive fish of age-1 up until the age of maturity, whereafter they became reproductive and reached their maximum size in the adult stage.

1 $$\left[ {\begin{array}{*{20}c} {Y_{{\left( {t + 1} \right)}} } \\ {SA_{{1\left( {t + 1} \right)}} } \\ \vdots \\ {A_{{n\left( {t + 1} \right)}} } \\ \end{array} } \right] = \left[ {\begin{array}{*{20}c} 0 & \cdots & 0 & {\frac{{f_{{A_{n} }} \cdot r_{Y} }}{2}} \\ {r_{{SA_{1} }} } & \cdots & 0 & 0 \\ \vdots & \cdots & \vdots & \vdots \\ 0 & \cdots & {r_{{A_{n} }} } & {m_{{A_{n} }} } \\ \end{array} } \right] \times \left[ {\begin{array}{*{20}c} {Y_{\left( t \right)} } \\ {SA_{1\left( t \right)} } \\ \vdots \\ {A_{n\left( t \right)} } \\ \end{array} } \right]$$

**Fig. 1:**
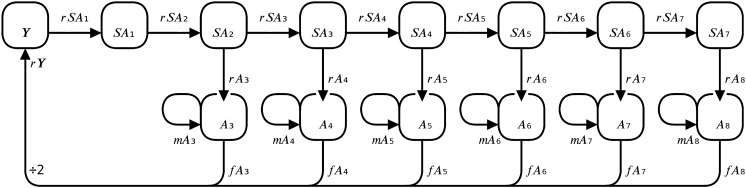
Flow diagram representing the age- and stage-structured population model of Bighead Carp. Depending on the maturation scenario considered, only the adult stage with the matching age-class (${A}_{n}$) will be active in a given simulation. Adults spawn to produce young-of-year $\left(Y\right)$ that develop into subadults $\left({SA}_{n}\right)$, and subsequently recruit to successive subadult age-classes until maturation into the adult stage. Each stage category differs in its survival function, and each age class differs in its size-at-age. These two factors together supply varying size-dependent survival probabilities for each individual stage in the model. Fecundities increase with age across adult age-classes, and are divided by two before young-of-year recruitment, as only female Bighead Carp are modelled. See Supplementary Table 7 for a description of state variables, and Supplementary Table 6 for parameter values

Only the adult age-class matching the age of maturity was active in a given simulation, while the number of open subadult age-classes could range from 2 to 7 (Fig. 1). Beginning at the young-of-year stage, age-0 individuals were recruited to successive subadult age-classes between 1 and 7 years (${r}_{S{A}_{n}}$). Upon reaching maturity, individuals were recruited to the adult stage (${r}_{{A}_{n}}$), and remained there until mortality occurred $\left({m}_{{A}_{n}}\right)$. All recruitment and mortality parameter values were derived from size-based, annual survival rates for each state variable. The parameter governing the persistence of adults was called adult mortality to differentiate it from other recruitment parameters despite having a similar composition.

Using a natural mortality model for juvenile and adult fishes (Eq. 2), where ${M}_{W}$ is the annual rate of natural mortality at weight $W$ in grams, ${M}_{u}$ is the natural mortality rate at unit weight, and $b$ is a life stage-specific allometric scaling factor (Lorenzen 1996), annual survival rates were calculated for young-of-year (Eq. 3a) and adults (Eq. 3c). Subadult survival was calculated by averaging these two functions (Eq. 3b) as parameters for this specific life stage were not given by Lorenzen (1996). To parameterize growth under each age of maturity, maturation data (Table 1) and von Bertalanffy growth functions, reported for Bighead Carp populations in various regions globally (Table 2), were retrieved from the literature and paired by their source location. These pairings supplied the average length-at-age (mm) values for subadult and adult age-classes across maturation scenarios; however, due to a lack of data on their size variation at different maturity ages, young-of-year were assumed to have a fixed size of 20 mm (Cuddington et al. 2014). Before being supplied to survival functions, length-at-age values were converted into weight (g) using Eq. 4, which was based on published Bighead Carp length–weight relationships (Cuddington et al. 2014). Adult fecundity was parameterized using the range of egg production estimates reported in Cuddington et al. (2014). Lower and upper range limits were assigned to age-3 and age-8 adults respectively, and remaining adult age-classes were assigned interpolated fecundity values, which either scaled linearly or logarithmically with the age of maturity. These two fecundity settings were used to explore the trade-off between maturation age and reproductive output under different exchange rates. In contrast to the conventional assumption that reproductive output scales isometrically with mass, logarithmic scaling was used to consider the empirically supported notion that larger fish are disproportionately more fecund (Barneche et al. 2018). The model was used to explore differences in population performance under different maturation schedules and, for simplicity, the effects of limited reproductive opportunity were not considered. Only female individuals were tracked within the model, and it was assumed that a single spawning event occurred per year and that neither mate availability nor sperm were limiting. The eggs produced each year $\left({f}_{{A}_{n}}\right)$ were accordingly divided by two, assuming an even sex ratio (Cuddington et al. 2014), before recruitment to the young-of-year stage (${r}_{Y}$).

2 $$M_{W} = M_{u} W^{b}$$

3a $$S_{Y} = 1 - \left( {1 - e^{{ - 2.7 \cdot W^{ - 0.315} }} } \right)$$

3b $$S_{SA} = 1 - \left( {1 - e^{{ - 3 \cdot W^{ - 0.288} }} } \right)$$

3c $$S_{A} = 1 - \left( {1 - e^{{ - 3.30 \cdot W^{ - 0.261} }} } \right)$$

4 $$W = 10^{{3.13 \cdot {\text{log}}_{10} L - 5.35}}$$

**Table 1:** Age of first maturity (in years) for female Bighead Carp in different locations around the world. Adapted from Cooke (2016)

Lat	Maturity	Location	References
13°N	≤ 2	Reared in cages in the Philippines	Santiago et al. (1991, 2004)
39°N	3	Missouri River, USA	Schrank and Guy (2002)
45°N	3–4	Central China	Kuronuma (1968) in Kolar et al. (2007)
NA	3–4	Sub-tropical and tropical climate	Woynarovich and Horváth (1980) in Kolar et al. (2007)
34°N	3–4	Arkansas aquaculture ponds	Henderson (1979) in Kolar et al. (2007)
22°N	4–5	China	Huet (1970)
48°N	5–6	Northeast China	Kuronuma (1968) in Kolar et al. (2007)
43°N	6	Terek region of Caspian basin	Abdusamadov (1987)
49°N	6–7	Romania	Huet (1970)
NA	6–8	Temperate climates	Woynarovich and Horváth (1980) in Kolar et al. (2007)
50°N	8–9	Kiev region	Huet (1970)

**Table 2:** Bighead Carp parameters for the Bertalanffy growth curve $({L}_{t}={L}_{\infty }\left(1-{e}^{-K\left(t-{t}_{0}\right)}\right))$, where ${L}_{t}$ is the body length at time $t$, ${L}_{\infty }$ is the maximum possible length, $K$ is a growth parameter, and ${t}_{0}$ is the time at which ${L}_{t}$ is zero

Latitude	Location	${L}_{\infty }$ (mm)	$K$	${t}_{0}$	References
30°N	Lake Donghu, China	1176	0.3088	0.5392	Jingrong (1986)
38°N	Middle Mississippi River, USA (1998)	1044	0.35	0.14	Nuevo et al. (2004)
38°N	Middle Mississippi River, USA (1999)	1093	0.28	0.098	Nuevo et al. (2004)
39°N	Missouri River, USA	774	0.491	0.43	Schrank and Guy (2002)
40°N	Biliuhe Reservoir, China	940.5	0.1915	0.04	Jiang et al. (1994)
42°N	Dahuofang Reservoir, China	915	0.2946	0.4849	Weiliang (1993)
45°N	Lake Katlabukh, Ukraine	1897.488	0.04001088	− 4.437552	Galina (1991) in Nuevo et al. (2004)
47°N	Kakhovka Reservoir, Ukraine	861.5957	0.279738	− 1.146815	Galina (1991) in Nuevo et al. (2004)
49°N	Kremenchug Reservoir, Ukraine	1025.008	0.1788562	− 0.4843691	Galina (1991) in Nuevo et al. (2004)
54°N	Lake Dgal Wielki, Poland	1098.695	0.1563503	0.2275022	Krzywosz et al. (1977) in Nuevo et al. (2004)

Listed parameters were either fitted from size-at-age data retrieved from the literature (Supplementary Table 12), or adapted from Table 4 in Cooke (2016). Location—year in parentheses represent year of observation if more than one year included in reference

### Growth rate, sensitivity, and elasticity analysis

Deterministic population growth rates ($\lambda$) were calculated for each maturation age and fecundity scaling setting (Supplementary Fig. 5) along with the sensitivity and elasticity of $\lambda$ to model parameters. Sensitivity and elasticity are metrics that have been used to identify suitable targets for management interventions (Caswell 2000). Sensitivity predicts the hypothetical impact on population growth from changes to a given vital rate (de Kroon et al. 1986), and elasticity is a proportional measure of sensitivity that allows for better comparison between parameters measured on different scales, such as recruitment and fecundity (Caswell 2000). Changes to parameters with the highest sensitivity or elasticity values would influence population growth more than the same change to any other parameter. The projected population growth rate was determined from the dominant eigenvalue of the matrix. The sensitivity ($S$) of each vital rate, which is the absolute response of $\lambda$ to additive changes in parameters, was calculated for each matrix element $a_{ij}$ as $S_{ij} = \frac{\partial \lambda }{{\partial a_{ij} }} = \frac{{\overline{v}_{i} w_{j} }}{{\left\langle {w,v} \right\rangle }}$. The denominator is the dot product of the right and left eigenvectors, which can be ignored when examining the relative sensitivities of $\lambda$ to matrix elements (Caswell 2001). Accordingly, the sensitivity of $\lambda$ to changes in ${a}_{ij}$ is equivalent to the product of the $i$th element of the reproductive value vector ($v$) and the $j$th element of the stable stage distribution ($w$). The elasticity ($E$) of matrix elements ${a}_{ij}$, the proportional response of $\lambda$ to proportional changes in parameter values, was calculated as $E_{ij} = \frac{{a_{ij} }}{\lambda }\frac{\partial \lambda }{{\partial a_{ij} }} = \frac{\partial \,\log \,\lambda }{{\partial \,\log \,a_{ij} }}$. All calculations were performed using the R statistical computing language (R Core Team 2020). Sensitivity outputs corresponding to impossible transitions between matrix elements were removed from analysis, leaving a corresponding number of outputs as the elasticity analysis, one per model parameter. Differences between growth rates, and the results of the sensitivity and elasticity analyses between scenarios, were compared to determine which parameters and life stages were most influential to population performance across various conditions.

### Establishment analysis

Populations arising from an introduction event were simulated under a range of scenarios to explore the effects of maturation schedule and founder ages (which determines reproductive output) in the early stages of invasion. All simulations began with a reproductive event between one female founder, an individual adult of variable reproductive output depending on its age of maturity, and at least one male assumed to be present. Founder scenarios involve different adult age-classes for the single female present at ${T}_{0}$, with the founder age indicating the maturation age of the founder and its corresponding fecundity and survival parameters. Different founder scenarios change the initial condition vector of the population but not the transition matrix (Eq. 1), so analytical solutions were not possible, and simulation was required. Populations were simulated for all combinations of founder and maturation ages, under each fecundity-scaling setting, resulting in two sets of 36 unique scenario simulations (Supplementary Fig. 5). Simulations were run until populations had recruited 1000 or more female adults (Tables 3, 4), which is an arbitrarily large endpoint taken from Cuddington et al. (2014), used as a simple representation of population establishment. The influence of different founder and maturation scenarios was examined by measuring the number of years it took for populations to establish. Comparison of population performance at one maturation age across founder scenarios showed the impact of the different reproductive abilities between founders, as the length of time before adult recruitment and reproduction was fixed. Alternatively, population performance among maturity ages was compared through examination of maturation scenarios under a fixed founder scenario.

**Table 3:** Establishment time (years until population has recruited ≥ 1000 adults) across all combinations of founder (${\text{Fn}}$) and maturity (${\text{Mn}}$) scenarios, under logarithmic-scaling fecundity. Bold numbers indicate establishment times where founder and maturity scenarios correspond to the same age

Scenario	M3	M4	M5	M6	M7	M8
F8	11	11	14	15	18	**27**
F7	14	15	18	21	**24**	27
F6	17	16	19	**21**	24	28
F5	21	20	**22**	22	25	30
F4	24	**22**	25	25	28	36
F3	**27**	25	26	29	32	37

**Table 4:** Establishment time for simulations with linear fecundity increases across maturation ages

Scenario	M3	M4	M5	M6	M7	M8
F8	11	10	12	14	17	**27**
F7	12	10	12	14	**17**	27
F6	13	10	12	**14**	17	27
F5	14	10	**12**	15	18	27
F4	17	**11**	13	16	24	28
F3	**27**	15	18	22	26	37

The number of years until the population has recruited ≥ 1000 adults are shown for all combinations of founder (${\text{F}}{\text{n}}$) and maturity (${\text{Mn}}$) scenarios, under linear-scaling fecundity. Bold numbers indicate establishment times where founder and maturity scenarios correspond to the same age

## Results

### Population growth rates

**Fig. 2:**
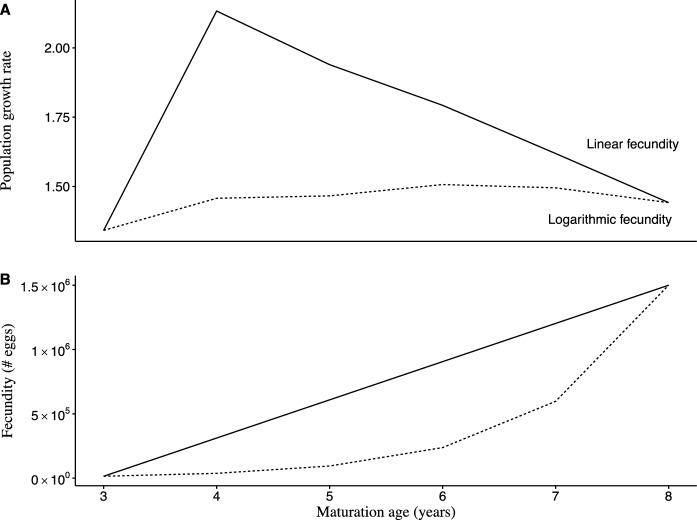
Multiplot of population growth rate ($\lambda$) and fecundity (number of eggs per adult female) at each maturation age under linear (solid line) and logarithmic-scaling (dashed line) fecundity settings

Growth rates followed a unimodal distribution under both logarithmic and linear-scaling fecundity settings, but their magnitude and rate of change across maturity ages differed (Fig. 2). For linear-scaling fecundity, maturation at age-4 produced the maximum population growth rate ($\lambda$ = 2.13), while maturation at age-6 produced the fastest growth under logarithmic scaling ($\lambda$ = 1.51). Both fecundity-scaling scenarios shared the lowest population growth rates, resulting from maturation at age-3, followed by maturation at age-8 (Table 5). The variation among growth rates for age-4 to age-7 maturation scenarios was 5 times greater under linear scaling ($\sigma$ = 0.3), compared to logarithmic scaling ($\sigma$ = 0.06). Growth rates changed less across maturation ages under logarithmic fecundity, differing by only $\pm$ 0.01 between some scenarios and were lower by 16% on average (Fig. 2a). Generally, linear scaling produced higher growth rates with a greater range of values, resulting in faster growth at younger ages of maturity, except for age-3. Alternatively, populations grew faster at middle ages of maturity when fecundity scaled logarithmically, but growth rates were lower overall, and did not change as much between ages of maturity.

**Table 5:** Deterministic growth rates $\left(\lambda \right)$ across maturity scenarios under logarithmic and linear-scaling fecundity

Scenario	Logarithmic	Linear
${\text{M3}}$	1.34	1.34
${\text{M4}}$	1.46	2.13
${\text{M5}}$	1.47	1.94
${\text{M6}}$	1.51	1.79
${\text{M7}}$	1.49	1.63
${\text{M8}}$	1.44	1.44

Growth rates were generally lower under logarithmic scaling, and peaked at age-6 maturation, whereas linear-scaling fecundity had its highest value at age-4

### Sensitivity of growth rate (${\varvec{\lambda}}$)

**Fig. 3:**
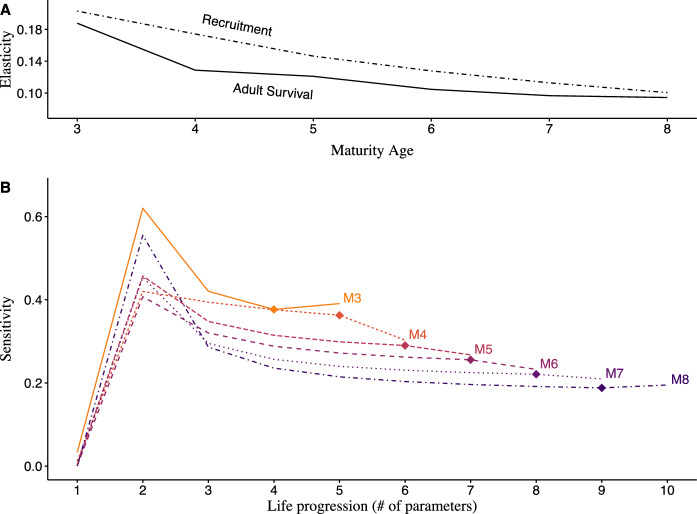
Line plots of elasticity (**A**) and sensitivity (**B**) of growth rate ($\lambda$) to all recruitment parameters and adult survival under logarithmic-scaling fecundity. **A** Elasticity of $\lambda$ to all recruitment parameters (dashed line) and adult survival (solid line) across maturation schedules. **B** Sensitivity of the population growth rate to every parameter in each maturity scenario (${\text{Mn}}$), ordered successively by consecutive life stage. Each maturity scenario line begins at young-of-year recruitment (${r}_{Y}$), and progresses though successive subadult recruitments until adult recruitment (marked by diamond) and adult survival thereafter. See Supplementary Table 10 for specific elasticity values

**Fig. 4:**
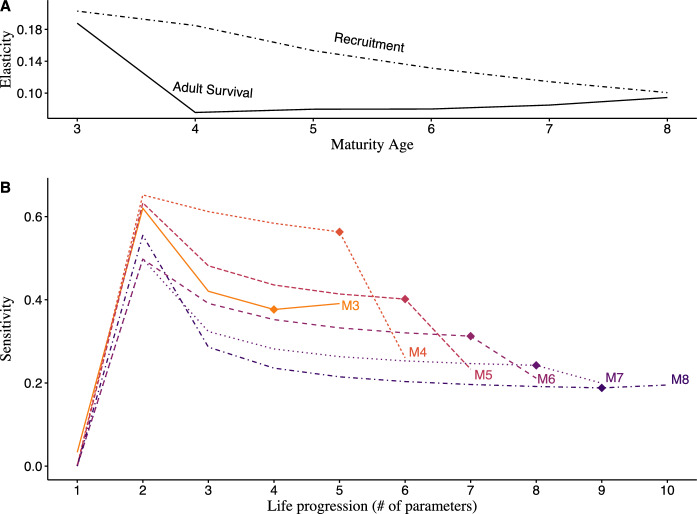
Line plots of elasticity (**A**) and sensitivity (**B**) of growth rate ($\lambda$) to all recruitment parameters and adult survival under linear-scaling fecundity. **A** Elasticity of $\lambda$ to all recruitment parameters (dashed line) and adult survival (solid line) across maturation schedules. **B** Sensitivity of the population growth rate to every parameter in each maturity scenario (${\text{Mn}}$), ordered successively by consecutive life stage. Each maturity scenario’s line begins at young-of-year recruitment (${r}_{Y}$), and progresses though successive subadult recruitments until adult recruitment (diamond marker) and adult survival thereafter. See Supplementary Table 11 for specific elasticity values

Overall, the population growth rate ($\lambda$) was least sensitive to young-of-year (age-0) recruitment, followed by adult survival, and adult recruitment (Figs. 3, 4). Population growth was most sensitive to age-1 recruitment and progressively less sensitive to the recruitment of older life stages. Most parameters had less influence on $\lambda$ in scenarios with older maturation ages. When maturation occurred at earlier ages, population growth was more sensitive to younger life stages and also more sensitive to each individual life stage, with the exception of age-1 recruitment (${r}_{S{A}_{1}}$).

While $\lambda$ was generally more sensitive to all life stages in younger maturation scenarios, the influence of age-1 recruitment changed non-linearly with the age of maturity. Age-1 recruitment was most influential when maturation occurred at age-3 and least influential at age-6 when fecundity scaled logarithmically (Supplementary Table 8). Under linear-scaling fecundity, age-1 recruitment was most influential at age-4 maturation and least at ages-6 and -7 (Fig. 4). Additionally, $\lambda$ was more sensitive to recruitment parameters in the age-4 and age-5 maturation scenarios than those in age-3 under linear-scaling fecundity compared to logarithmic scaling (Supplementary Table 9). In this case, the trend where parameters decreased in influence across increasing maturation ages began only after age-3. This occurred because $\lambda$, under linear-scaling fecundity, was 12% more sensitive to recruitment and 5% less to adult survival on average, with a greater degree of change occurring in younger maturation scenarios. For example, population growth in the age-4 maturation scenario became 26% more sensitive to general recruitment and 10% less to adult survival compared to when fecundity scaled logarithmically. Overall, $\lambda$ was consistently more sensitive to recruitment than adult survival for age-4 to age-7 maturation scenarios, but this disparity was larger at younger ages of maturity and increased overall when fecundity scaled linearly. Conversely, in the age-3 and age-8 maturation scenarios, $\lambda$ was slightly more sensitive (+ 0.01) to adult survival than adult recruitment.

### Elasticity of population growth rate (${\varvec{\lambda}}$)

Across all scenarios, the elasticity of the population growth rate ($\lambda$) to recruitment was greater than that for adult survival, but declined in value with increasing age of maturity (Supplementary Tables 10, 11). The elasticity of $\lambda$ to adult survival declined similarly across maturation ages when fecundity scaled logarithmically and was least influential at age-8 maturation (Fig. 3). Under linear scaling, population growth was least influenced by adult survival at age-4 maturation, with small increases across subsequent maturation scenarios (Fig. 4). Additionally, proportional changes in adult survival contributed considerably less to population growth overall; compared to the outcomes produced under logarithmic fecundity, the average disparity in elasticity of $\lambda$ between parameters was 2.25 times larger. While the influence of adult survival to population growth was consistently less than that of recruitment, influence upon $\lambda$ was more evenly distributed among parameters under logarithmic fecundity. In this setting, recruitment and adult survival contributed to population growth in more similar proportions, with the mean influence of recruitment across maturation ages being 14% greater than that of adult survival. Conversely, when fecundity scaled linearly, recruitment contributed 30% more than adult survival on average.

### Establishment time

In scenarios where founders matured at the same age as their descendants, establishment occurred in the least amount of time for the maturation scenarios with the highest growth rates. Establishment occurred in 21 years under age-6 maturation for logarithmic fecundity (Table 3), and 11 years under age-4 maturation when fecundity scaled linearly (Table 4). Additionally, there was a greater range of outcomes under linear-scaling fecundity ($\sigma$ = 7.27) compared to logarithmic fecundity ($\sigma$ = 2.64) in these scenarios. However, combinations of founder scenarios of different ages from the maturation scenario produced different results. In these scenarios, the fecundity of the individual female at the start of the simulation could be higher or lower than the fecundity of subsequently recruited adults. The variation across all outcomes under linear ($\sigma$ = 6.85) and logarithmic fecundity settings ($\sigma$ = 6.34) were more similar, but the relative impacts of each scenario were different.

Maturation at age-8 and an age-3 founder, resulted in an establishment time of 37 years, the maximum across all scenarios. Under logarithmic fecundity, the inverse combination of scenarios, maturation at age-3 and -4 combined with an age-8 founder, produced the minimum establishment time of 11 years (Table 3). When early-maturing adults produced considerably fewer eggs, founder age had a larger influence on establishment, which could overcome the disproportionate change in fecundity across maturation ages. In this case, fast-maturing, but less fecund, populations established over three times faster than highly fecund, late-maturing populations, when started by founders of the highest and lowest ages, respectively (Table 3).

Under linear-scaling fecundity, a minimum establishment time of 10 years occurred with age-4 maturation, across founder ages 5 to 8 (Table 4). In this case, establishment did not occur any sooner when the founder’s fecundity exceeded that of an age-5 adult (609 000 eggs). Similar thresholds were also present in each maturation scenario; establishment under scenarios with founders of ages 5 to 8 were generally closer in value compared to scenarios under ages 3 and 4. Additionally, outcomes produced by age-4 founders were only 1–2 years greater than the minimum in most maturation scenarios. When fecundity increased at a consistent rate across maturation ages, older, more fecund founders were not necessary to produce rapid establishment. When fecundity was reduced in younger adults, older founders were more influential to establishment.

Generally, similar establishment outcomes could be achieved across founder scenarios when fecundity scaled linearly (Supplementary Fig. 6). When younger maturation was comparatively less penalized by fecundity, the age of maturity was more impactful to establishment. Under logarithmic fecundity, establishment was more affected by founder age than maturation age; however, outcomes also varied more evenly across both scenario types (Supplementary Fig. 7). There was less difference between the relative influence of founder and maturation scenarios when late-maturing adults were disproportionately more fecund.

## Discussion

Although Bighead Carp population performance (growth rate and establishment time) was affected by multiple factors controlled by maturity (fecundity, survival, and generation time), certain life stages were consistently more impactful regardless of maturation age. Across all scenarios, population growth was affected most by age-1 recruitment, least by young-of-year recruitment, and less by adult mortality than recruitment as a whole. Population growth rates were higher in scenarios where adult mortality had less, or overall recruitment had greater, influence. The highest population growth rates occurred at intermediate ages of maturity (e.g. 4–6) and skewed towards younger maturation ages when fecundity scaled linearly, and older ages when scaled logarithmically. The optimal balance between generation time, reproductive output, and early-life survival enables greater population growth by increasing the number of individuals that reproduce at least once. Changes to fecundity may be more influential to population growth than the two other factors controlled by maturation. Alternatively, age of maturity was the primary influence on establishment irrespective of fecundity—establishment time was shortest under age-4 maturation in 70 of 72 scenarios. Founder age also affected establishment time, but primarily for younger maturation ages under logarithmic scaling fecundity. Only in scenarios where fecundity was diminished by early maturity were older founders with higher reproductive output able to further reduce establishment times. However, when fecundity scaled linearly with maturation age founders that matured at ages older than 4 had little to no additional impact, suggesting that there is a threshold value of eggs (38 000 in our model) above which fecundity is far less limiting to establishment time.

### Recruitment

The population growth rate was most sensitive to age-1 recruitment and progressively less sensitive to each recruitment parameter thereafter, indicating that changes in recruitment impacted population growth less at older life stages. Other population models for invasive xenocypridid carps have also shown population growth to be most sensitive to the recruitment of early life stages (Cuddington et al. 2014) and pointed to the survival of early life stages as critical for establishment (Jones et al. 2017; Smyth and Drake 2022). Our results exhibited a similar pattern with the exception of eggs and larvae; young-of-year recruitment appeared to be the least influential to population growth based on the sensitivity analysis. This contrasts with other studies that have shown Bighead Carp population growth to be most sensitive to juvenile (eggs, larvae, age-0) survivorship in both deterministic (Cudmore et al. 2012) and stochastic (Cuddington et al. 2014) models. However, the young-of-year recruitment parameter in our study encompassed more processes than the other parameters, incorporating both fecundity and survival values. This difference makes direct comparisons to other parameters misleading because fecundity values can be arbitrarily large while survival and recruitment values range from 0 to 1 (Caswell 2000). Thus, the survival of eggs and larvae could still potentially be highly impactful but misinterpreted when examining sensitivity alone. The elasticity analysis showed that changes to young-of-year recruitment would affect population growth no less than any other vital rate changed by an equivalent proportion. Additionally, the disparity between adult survival and recruitment shown by the elasticity analysis reveals a pattern of older individuals contributing less to population growth, which is consistent with other studies that have found population growth to be most sensitive to the juvenile life stage followed by age-1 (Cuddington et al. 2014; Cudmore et al. 2012).

Adult survival was consistently less impactful than recruitment across maturation ages with greater difference occurring at younger ages of maturity and when fecundity was not enhanced by maturing later. This finding suggests that the first reproduction of new generations contributes more to population growth than the continued reproduction of existing adults, and that populations are less affected by adult mortality at higher levels of reproductive output.

### Effects of fecundity scaling

Under logarithmic-scaling fecundity, population growth rates peaked at age-6 maturation, versus age-4 under linear scaling, demonstrating that differences in reproductive output can influence which maturation schedule is optimal. Delayed maturation results in longer generation times that are well known to limit population growth (Birch 1948; Pianka 1976), but can be compensated by increased reproductive output (Cole 1954). However, performance differences between scaling scenarios show that linear increases in fecundity are not commensurate to the drawbacks of delayed maturation. Population growth rates under logarithmic scaling were more similar in value between maturation ages compared to those under linear scaling, suggesting that the effect of delayed maturation on population growth is also non-linear. Therefore, progressively greater increases in fecundity are required to sufficiently offset each additional year of development. It is unclear how much fecundity differs for larger and older Bighead Carp, but reproductive output in fishes can be greatly underestimated when assumed to scale isometrically with mass (Barneche et al. 2018). Presumably, population growth rates under linear scaling fecundity reflect an exaggerated advantage to early maturity. Comparing the performance of individual maturation schedules between scaling scenarios also reveals that increased fecundity has a greater effect on population growth at younger ages of maturity, but changes in fecundity between maturation ages could involve differences in both quantity and quality of eggs. In other freshwater fishes, larger and older females produce more eggs (Baccante and Reid 1988), but egg survival is also positively correlated with maternal size and age (Johnston 1997). This may happen because larger female fishes not only produce eggs in greater quantities but with higher energy content as well, leading to larger offspring that survive better (Barneche et al. 2018). By considering the potential improvements in young-of-year recruitment in addition to larger clutch size, the consequences of adult size and age may further balance the impact of increased generation time across maturation schedules.

### Founder and maturation age

Maturation age affects multiple population characteristics but the most influential difference that impacts establishment is generation time. Assuming survival is related to size for Bighead Carp (Cuddington et al. 2014) and that larger female fishes produce more eggs (Barneche et al. 2018), changes in maturation age would also affect fecundity and recruitment. Other studies modelling invasive xenocypridid carps have found that changes to recruitment can have disproportionate impacts on the likelihood of establishment (Smyth and Drake 2022), yet changes in maturation schedule do not affect their ability to establish successfully despite slower population growth rates at later ages of maturity (Jones et al. 2017). The explanation could be that size-at-age differences between maturation schedules, and the resulting effects on mortality, are not large enough to significantly impact population growth and establishment. Instead, the primary effect of maturation age on establishment was generation time—age-4 maturation produced a faster establishment time than most simulations regardless of increased fecundity or survival rates. Early maturity is well known to increase population growth rates for starting populations (Stearns 1992). Despite population growth rates being higher at older ages of maturity under logarithmic scaling, generation time was still more influential to establishment by increasing population growth in the early years after introduction. The contribution of older, more fecund founders also had a limited effect on establishment time when fecundity was not diminished by early maturation, but increasing founder age produced a greater difference for early maturation ages with lower fecundities. Accordingly, generation time was more impactful than an increased number of second-generation propagules. This is consistent with life-history theory, which proposes that fishes that mature earlier and produce smaller clutches of fast-growing offspring are less vulnerable to high adult mortality and are well-equipped to achieve high rates of population growth quickly (Winemiller and Rose 1992).

### Implications for species invasion

There is little chance for eradication once aquatic invasive species have established (Leung et al. 2002), so it is critical to take preventative measures and develop methods to pre-emptively assess the threat of potential invaders (Chen et al. 2007). Understanding population sensitivity to various life stages is foundational to the development of successful control strategies and the technology required to execute them. Our study demonstrated that shorter generation times increase population growth more than greater fecundity in the short term, and perturbations to age-1 recruitment have the greatest impact on population growth. These results have important implications for management, indicating that populations that mature at earlier ages present a greater probability of establishment and that targeting small-bodied individuals from early life stages is critical for population control. While the high sensitivity of population growth to early life stages highlights them as a desirable management target, they might not be a practical control target if it is difficult to influence their vital rates (Caswell 2000). High densities and smaller sizes of younger individuals in invasive xenocypridid carp populations make removal both difficult and costly (Garvey et al. 2007), and most management interventions currently focus on the removal of adults. While a lack of suitable field strategies makes targeting early life stages difficult with current control methods, our results show the critical need for technological development that could enable successful management of age-1 individuals in the future. Understanding population sensitivity to stage-specific removals can also identify unsuitable targets. While some ecological management objectives can still be achieved by suppressing populations in lieu of eradication, this could actually be counterproductive when the target species has compensatory population growth (Prior et al. 2018). If the wrong life stages are targeted, or certain life stages are exploited to the wrong degree, increased mortality could increase population growth by freeing the remaining individuals from intraspecific competition (Ricker 1975). Silver Carp modelling has shown that the population production of biomass can increase under greater levels of exploitation, particularly when individuals of intermediate sizes are targeted (Garvey et al. 2007). Similar concerns have been expressed for the management of Sea Lamprey (*Petromyzon marinus*) in the Great Lakes; however, compensatory mechanisms following population control have been found to have limited influence compared to density-independent variation in recruitment (Jones et al. 2003). The management of each life stage of Bighead Carp varies in difficulty and cost, regardless of their respective contribution to population growth. While the results of the sensitivity analyses suggest that age-1 is the most impactful life stage to target because changes to their survival affect population growth by the largest amount, practical opportunities to reduce age-1 abundance may be limited. Accordingly, our findings do not advocate for the exclusive removal of specific life stages, but emphasize that the relative effect of removal among age classes should be considered first before attempting control on all life stages. The development of successful control strategies requires knowledge of the effects of stage-specific removals on population trajectory, the feasibility of targeting influential life stages, and the methods or tools that could enable such management interventions.

### Model limitations

We designed our scenarios to allow for simple comparisons of baseline population performance between maturation schedules as a proxy of thermal conditions; therefore, our findings do not account for vital-rate differences beyond potential climate effects, such as local resource and habitat availability. Differences in performance were evaluated in the absence of potential mate limitation and all simulations were initiated with a single breeding pair. Additionally, single-population deterministic models assume that demographic rates are essentially constant (Beissinger and Westphal 1998), and although we used a deterministic model to facilitate comparison among scenarios, the estimated times until establishment would show substantially greater variation if stochasticity was incorporated into the modelling. Density dependence was also not considered in our population simulations; however, we assumed density dependence to have a negligible effect as the model was used to explore the limited timeframe between a potential introduction and establishment.

### Conclusions

Our results indicate that population performance is impacted more by recruitment than adult mortality and that younger life stages are the most influential. Age-1 recruitment was determined to have the greatest impact on population growth, and adult mortality had the second to lowest impact. While our results show that young-of-year recruitment had the least influence on population growth, this is a potentially misleading result due to the incorporation of fecundity into the young-of-year recruitment parameter; other studies have pointed to young-of-year as the most influential life stage for invasive xenocypridid carps (Cuddington et al. 2014; Cudmore et al. 2012; Jones et al. 2017; Smyth and Drake 2022). Population growth was lowest for the youngest and oldest maturation schedules, suggesting that the optimal balance of generation time and sufficient fecundity was achieved at intermediate ages of maturity. The scenarios with the highest population growth were also those least affected by changes to adult mortality. However, despite potentially limiting population growth, shorter generation times under earlier ages of maturity mattered the most for establishment, and maturing at age-4 resulted in the shortest establishment time in the vast majority of scenarios. While suppressing certain life stages would appear to be more important because they are more impactful to population growth, it may not actually be practical or particularly beneficial to target them. Applying too much control could inadvertently increase population growth, and young life stages can be too difficult and expensive to capture due to their small size and high abundance in the wild. Anticipating the age of maturity of an invading population can help when estimating how quickly establishment could occur, and increasing temperatures expected under climate change suggest that the rate of potential establishment would increase in coming years. However, climate change also involves several other environmental factors, such as more variable precipitation, which could increase the frequency of reproductive events and broaden the spawning season. This increased window of opportunity for reproduction may not necessarily be utilized, and could even be disadvantageous if it results in poor timing between growth and development with resource availability or other phenological mismatches. Nonetheless, the model presented here helps to provide a foundation for better understanding these factors in the future.

### Supplementary Information

Below is the link to the electronic supplementary material.

Supplementary file1 (DOCX 128 KB)

## Data Availability

Data underlying this manuscript are provided in the supplementary material.
