# Supplementary material for: Non-linear effects on the population performance of Bighead Carp under different maturation schedules
Source: Biol Invasions. 2023 Aug 12;25(11):3567–81. doi: 10.1007/s10530-023-03126-z (PMC10514160; doi:10.1007/s10530-023-03126-z)
Supplement: Supplementary file 1 — Supplementary file1 (DOCX 128 KB) [file 10530_2023_3126_MOESM1_ESM.docx]

# Supplementary

Equation (1) represents a stage structured population matrix describing the population growth of Bighead Carp. The number of individuals in each stage and age-class at time $t+1$ (left vector), is the product of the recruitment rates of the transition matrix (centre), and the demographics at time $t$ (right vector).

$$\left[ \begin{matrix} Y_{\left( t+1 \right)} \\ {SA_{1}}_{\left( t+1 \right)} \\ {SA_{2}}_{\left( t+1 \right)} \\ {SA_{3}}_{\left( t+1 \right)} \\ {SA_{4}}_{\left( t+1 \right)} \\ {SA_{5}}_{\left( t+1 \right)} \\ {SA_{6}}_{\left( t+1 \right)} \\ {SA_{7}}_{\left( t+1 \right)} \\ {A_{3}}_{\left( t+1 \right)} \\ {A_{4}}_{\left( t+1 \right)} \\ {A_{5}}_{\left( t+1 \right)} \\ {A_{6}}_{\left( t+1 \right)} \\ {A_{7}}_{\left( t+1 \right)} \\ {A_{8}}_{\left( t+1 \right)} \end{matrix} \right]=\left[ \begin{matrix} 0 & 0 & 0 & 0 & 0 & 0 & 0 & 0 & \frac{f_{A_{3}}\cdot r_{Y}}{2} & \frac{f_{A_{4}}\cdot r_{Y}}{2} & \frac{f_{A_{5}}\cdot r_{Y}}{2} & \frac{f_{A_{6}}\cdot r_{Y}}{2} & \frac{f_{A_{7}}\cdot r_{Y}}{2} & \frac{f_{A_{8}}\cdot r_{Y}}{2} \\ r_{SA_{1}} & 0 & 0 & 0 & 0 & 0 & 0 & 0 & 0 & 0 & 0 & 0 & 0 & 0 \\ 0 & r_{SA_{2}} & 0 & 0 & 0 & 0 & 0 & 0 & 0 & 0 & 0 & 0 & 0 & 0 \\ 0 & 0 & r_{SA_{3}} & 0 & 0 & 0 & 0 & 0 & 0 & 0 & 0 & 0 & 0 & 0 \\ 0 & 0 & 0 & r_{SA_{4}} & 0 & 0 & 0 & 0 & 0 & 0 & 0 & 0 & 0 & 0 \\ 0 & 0 & 0 & 0 & r_{SA_{5}} & 0 & 0 & 0 & 0 & 0 & 0 & 0 & 0 & 0 \\ 0 & 0 & 0 & 0 & 0 & r_{SA_{6}} & 0 & 0 & 0 & 0 & 0 & 0 & 0 & 0 \\ 0 & 0 & 0 & 0 & 0 & 0 & r_{SA_{7}} & 0 & 0 & 0 & 0 & 0 & 0 & 0 \\ 0 & 0 & r_{A_{3}} & 0 & 0 & 0 & 0 & 0 & m_{A_{3}} & 0 & 0 & 0 & 0 & 0 \\ 0 & 0 & 0 & r_{A_{4}} & 0 & 0 & 0 & 0 & 0 & m_{A_{4}} & 0 & 0 & 0 & 0 \\ 0 & 0 & 0 & 0 & r_{A_{5}} & 0 & 0 & 0 & 0 & 0 & m_{A_{5}} & 0 & 0 & 0 \\ 0 & 0 & 0 & 0 & 0 & r_{A_{6}} & 0 & 0 & 0 & 0 & 0 & m_{A_{6}} & 0 & 0 \\ 0 & 0 & 0 & 0 & 0 & 0 & r_{A_{7}} & 0 & 0 & 0 & 0 & 0 & m_{A_{7}} & 0 \\ 0 & 0 & 0 & 0 & 0 & 0 & 0 & r_{A_{8}} & 0 & 0 & 0 & 0 & 0 & m_{A_{8}} \end{matrix} \right]\times\left[ \begin{matrix} Y_{\left( t \right)} \\ {SA_{1}}_{\left( t \right)} \\ {SA_{2}}_{\left( t \right)} \\ {SA_{3}}_{\left( t \right)} \\ {SA_{4}}_{\left( t \right)} \\ {SA_{6}}_{\left( t \right)} \\ {SA_{7}}_{\left( t \right)} \\ {SA_{5}}_{\left( t \right)} \\ {A_{3}}_{\left( t \right)} \\ {A_{4}}_{\left( t \right)} \\ {A_{5}}_{\left( t \right)} \\ {A_{6}}_{\left( t \right)} \\ {A_{7}}_{\left( t \right)} \\ {A_{8}}_{\left( t \right)} \end{matrix} \right]$$

######

**Table 6**. Values for parameters used in the Bighead Carp Population model under age-3 to age-8 maturation schedules. Model parameters include recruitment to young-of-year ($r_{Y}$), subadult ($r_{{SA}_{n}}$), and adult ($r_{A_{n}}$) age-classes, adult mortality ($m_{A_{n}}$), and fecundity ($f_{A_{n}}$). Logarithmically scaled fecundity values are differentiated from linear ones with an asterisk${}^{*}$ for age-4 to -7 adults.

|  | $\boldsymbol{r}_{\boldsymbol{Y}}$ | $\boldsymbol{r}_{\boldsymbol{S}\boldsymbol{A}_{\boldsymbol{1}}}$ | $\boldsymbol{r}_{\boldsymbol{S}\boldsymbol{A}_{\boldsymbol{2}}}$ | $\boldsymbol{r}_{\boldsymbol{S}\boldsymbol{A}_{\boldsymbol{3}}}$ | $\boldsymbol{r}_{\boldsymbol{S}\boldsymbol{A}_{\boldsymbol{4}}}$ | $\boldsymbol{r}_{\boldsymbol{S}\boldsymbol{A}_{\boldsymbol{5}}}$ | $\boldsymbol{r}_{\boldsymbol{S}\boldsymbol{A}_{\boldsymbol{6}}}$ | $\boldsymbol{r}_{\boldsymbol{S}\boldsymbol{A}_{\boldsymbol{7}}}$ | $\boldsymbol{r}_{\boldsymbol{A}_{\boldsymbol{n}}}$ | $\boldsymbol{m}_{\boldsymbol{A}_{\boldsymbol{n}}}$ | $\boldsymbol{f}_{\boldsymbol{A}_{\boldsymbol{n}}}$ |
| --- | --- | --- | --- | --- | --- | --- | --- | --- | --- | --- | --- |
| Age-3 | $10.9\times{10}^{-4}$ | 0.44 | 0.65 | - | - | - | - | - | 0.72 | 0.64 | $1.5\times{10}^{4}$ |
| Age-4 | $10.9\times{10}^{-4}$ | 0.60 | 0.64 | 0.68 | - | - | - | - | 0.70 | 0.62 | 3.8${}^{*} \vert$ $31\times{10}^{4}$ |
| Age-5 | $10.9\times{10}^{-4}$ | 0.47 | 0.62 | 0.68 | 0.72 | - | - | - | 0.74 | 0.66 | 9.5${}^{*} \vert$ 6$1\times{10}^{4}$ |
| Age-6 | $10.9\times{10}^{-4}$ | 0.47 | 0.6 | 0.67 | 0.71 | 0.74 | - | - | 0.75 | 0.68 | 24${}^{*} \vert$ 9$1\times{10}^{4}$ |
| Age-7 | $10.9\times{10}^{-4}$ | 0.37 | 0.57 | 0.66 | 0.7 | 0.73 | 0.75 | - | 0.76 | 0.69 | 60${}^{*} \vert120\times{10}^{4}$ |
| Age-8 | $10.9\times{10}^{-4}$ | 0.26 | 0.51 | 0.62 | 0.68 | 0.71 | 0.74 | 0.76 | 0.77 | 0.70 | $150\times{10}^{4}$ |

**Table 7**. State variables tracked by the population model, representing young-of-year, subadult, and adult life stages of varying age-classes. Depending on the maturation scenario considered, adults can mature between the ages of 3 and 8, and subadults accordingly can range in age from 1 to 7 years.

| Variable | Description |
| --- | --- |
| $Y$ | Young-of-year (Year-0) |
| ${SA}_{1}$ | Year-1 Subadults |
| ${SA}_{2}$ | Year-2 Subadults |
| ${SA}_{3}$ | Year-3 Subadults |
| ${SA}_{4}$ | Year-4 Subadults |
| ${SA}_{5}$ | Year-5 Subadults |
| ${SA}_{6}$ | Year-6 Subadults |
| ${SA}_{7}$ | Year-7 Subadults |
| $A_{3}$ | Year-3 Adults |
| $A_{4}$ | Year-4 Adults |
| $A_{5}$ | Year-5 Adults |
| $A_{6}$ | Year-6 Adults |
| $A_{7}$ | Year-7 Adults |
| $A_{8}$ | Year-8 Adults |

**Table 8**. Sensitivity of population growth rate ($\lambda$) to all model parameters across maturation scenarios, under logarithmic fecundity scaling. Larger sensitivity values indicate greater influence upon the total population growth rate per unit change for a specific parameter value.

|  | $\boldsymbol{r}_{\boldsymbol{Y}}$ | $\boldsymbol{r}_{\boldsymbol{S}\boldsymbol{A}_{\boldsymbol{1}}}$ | $\boldsymbol{r}_{\boldsymbol{S}\boldsymbol{A}_{\boldsymbol{2}}}$ | $\boldsymbol{r}_{\boldsymbol{S}\boldsymbol{A}_{\boldsymbol{3}}}$ | $\boldsymbol{r}_{\boldsymbol{S}\boldsymbol{A}_{\boldsymbol{4}}}$ | $\boldsymbol{r}_{\boldsymbol{S}\boldsymbol{A}_{\boldsymbol{5}}}$ | $\boldsymbol{r}_{\boldsymbol{S}\boldsymbol{A}_{\boldsymbol{6}}}$ | $\boldsymbol{r}_{\boldsymbol{S}\boldsymbol{A}_{\boldsymbol{7}}}$ | $\boldsymbol{r}_{\boldsymbol{A}_{\boldsymbol{n}}}$ | $\boldsymbol{m}_{\boldsymbol{A}_{\boldsymbol{n}}}$ |
| --- | --- | --- | --- | --- | --- | --- | --- | --- | --- | --- |
| $\text{M3}$ | 0.03 | 0.62 | 0.42 | - | - | - | - | - | 0.38 | 0.39 |
| $\text{M4}$ | 0.01 | 0.42 | 0.39 | 0.38 | - | - | - | - | 0.36 | 0.3 |
| $\text{M5}$ | 0.00 | 0.46 | 0.35 | 0.31 | 0.30 | - | - | - | 0.29 | 0.27 |
| $\text{M6}$ | 0.00 | 0.41 | 0.32 | 0.29 | 0.27 | 0.26 | - | - | 0.26 | 0.23 |
| $\text{M7}$ | 0.00 | 0.45 | 0.30 | 0.26 | 0.24 | 0.23 | 0.22 | - | 0.22 | 0.21 |
| $\text{M8}$ | 0.00 | 0.56 | 0.29 | 0.24 | 0.21 | 0.20 | 0.20 | 0.19 | 0.19 | 0.2 |

######

**Table 9**. Sensitivity of population growth rate ($\lambda$) to all model parameters across maturation scenarios, assuming linear increase in fecundity across maturation ages.

|  | $\boldsymbol{r}_{\boldsymbol{Y}}$ | $\boldsymbol{r}_{\boldsymbol{S}\boldsymbol{A}_{\boldsymbol{1}}}$ | $\boldsymbol{r}_{\boldsymbol{S}\boldsymbol{A}_{\boldsymbol{2}}}$ | $\boldsymbol{r}_{\boldsymbol{S}\boldsymbol{A}_{\boldsymbol{3}}}$ | $\boldsymbol{r}_{\boldsymbol{S}\boldsymbol{A}_{\boldsymbol{4}}}$ | $\boldsymbol{r}_{\boldsymbol{S}\boldsymbol{A}_{\boldsymbol{5}}}$ | $\boldsymbol{r}_{\boldsymbol{S}\boldsymbol{A}_{\boldsymbol{6}}}$ | $\boldsymbol{r}_{\boldsymbol{S}\boldsymbol{A}_{\boldsymbol{7}}}$ | $\boldsymbol{r}_{\boldsymbol{A}_{\boldsymbol{n}}}$ | $\boldsymbol{m}_{\boldsymbol{A}_{\boldsymbol{n}}}$ |
| --- | --- | --- | --- | --- | --- | --- | --- | --- | --- | --- |
| $\text{M3}$ | 0.03 | 0.62 | 0.42 | - | - | - | - | - | 0.38 | 0.39 |
| $\text{M4}$ | 0.01 | 0.65 | 0.61 | 0.58 | - | - | - | - | 0.56 | 0.26 |
| $\text{M5}$ | 0.00 | 0.63 | 0.48 | 0.44 | 0.41 | - | - | - | 0.4 | 0.23 |
| $\text{M6}$ | 0.00 | 0.50 | 0.39 | 0.35 | 0.33 | 0.32 | - | - | 0.31 | 0.21 |
| $\text{M7}$ | 0.00 | 0.50 | 0.32 | 0.28 | 0.26 | 0.25 | 0.25 | - | 0.24 | 0.2 |
| $\text{M8}$ | 0.00 | 0.56 | 0.29 | 0.24 | 0.21 | 0.20 | 0.20 | 0.19 | 0.19 | 0.2 |

**Table 10**. Elasticity of growth rate ($\lambda$) to recruitment ($r_{X_{n}}$) parameters, and adult mortality ($m_{A_{n}}$) across all maturation scenarios, under logarithmic-scaling fecundity. Stage recruitment is the elasticity value for each individual recruitment parameter, and total recruitment is the sum value for all transitions (stage recruitment multiplied by the number of stages in a given maturity scenario).

| Scenario | Stage recruitment | Total recruitment | Adult mortality |
| --- | --- | --- | --- |
| $\text{M3}$ | 0.203 | 0.812 | 0.188 |
| $\text{M4}$ | 0.174 | 0.87 | 0.130 |
| $\text{M5}$ | 0.147 | 0.88 | 0.121 |
| $\text{M6}$ | 0.128 | 0.895 | 0.105 |
| $\text{M7}$ | 0.113 | 0.903 | 0.097 |
| $\text{M8}$ | 0.101 | 0.906 | 0.094 |

**Table 11**. Elasticity of growth rate ($\lambda$) to recruitment ($r_{X_{n}}$) parameters, and adult mortality ($m_{A_{n}}$) across all maturation scenarios, assuming linear increase in fecundity across maturation ages. Stage recruitment is the elasticity value for each individual recruitment parameter, and total recruitment is the sum value for all transitions (stage recruitment multiplied by the number of stages in a given maturity scenario).

| Scenario | Stage recruitment | Total recruitment | Adult mortality |
| --- | --- | --- | --- |
| $\text{M3}$ | 0.203 | 0.812 | 0.188 |
| $\text{M4}$ | 0.185 | 0.924 | 0.076 |
| $\text{M5}$ | 0.153 | 0.920 | 0.080 |
| $\text{M6}$ | 0.131 | 0.920 | 0.080 |
| $\text{M7}$ | 0.114 | 0.915 | 0.085 |
| $\text{M8}$ | 0.101 | 0.905 | 0.094 |

**Table 12**. Mean back-calculated lengths at age (mm) for Bighead Carp from several locations. Lengths from Schrank and Guy (2002) were specifically for females; values from all other studies did not specify sex. These values were fitted to Von Bertalanffy growth functions in (Table 2).

| Location | Age-1 | Age-2 | Age-3 | Age-4 | Age-5 | Age-6 | Age-7 | Age-8 | Age-9 | Age-10 | Citation |
| --- | --- | --- | --- | --- | --- | --- | --- | --- | --- | --- | --- |
| Missouri  River (USA) | 189 | 354 | 541 | 665 | 746 | 857 | 690 | - | - | - | Schrank and Guy (2002) |
| Kakhovka Reservoir (Ukraine) | 389 | 472 | 586 | 682 | 741 | 771 | 762 | - | - | - | Galina (1991) |
| Lake  Dgal Wielki (Poland) | 125 | 242 | 392 | 503 | 580 | 667 | 716 | - | - | - | Krzywosz et al. (1977) |
| Kremenchug Reservoir (Ukraine) | 239 | 374 | 470 | 565 | - | 701 | 746 | 800 | - | - | Galina (1991) |
| Lake  Katlabukh (Ukraine) | 371 | 465 | 502 | 538 | 563 | 645 | 665 | 713 | 785 | 835 | Galina (1991) |

#

**Figure 5**. Visual representation of all scenario combinations examined in this study. Scenarios are composed of three categories, which are identified by the uppermost labels: type of fecundity change across adult age-classes, age of maturity, and age of population founder. Analysis of establishment time involved all three scenario categories. Analysis of growth rate ($\lambda$), and the sensitivity, and elasticity of ($\lambda$) to model parameters involved only fecundity, and maturity scenario settings.

######

**Figure 6**. Establishment times (years) under varying founder and maturation scenarios, assuming linear increase in fecundity. See Table 4 for the specific number of years until establishment.

**Figure 7**. Establishment times (years) under varying founder and maturation scenarios, under logarithmic-scaling fecundity. See Table 3 for the specific number of years until establishment.

**Figure 8**. Establishment times (years until 1000 or more adults are recruited) under all combination of maturity and founder scenarios, under logarithmic-scaling fecundity. Plot A shows founder scenarios as lines across the maturity age axis, while plot B shows maturity scenarios as lines across the founder age axis. See Table 3 for the specific number of years until establishment.

**Figure 9**. Plots of establishment times (years until 1000 or more adults are recruited) under all combination of maturity and founder scenarios, assuming linear increases in fecundity across maturation scenarios. Plot A shows founder scenarios as lines across the maturity ages, while plot B shows maturity scenarios as lines across founder ages. See Table 3 for the specific number of years until establishment.

# 
